# Fast Responsive, Reversible Colorimetric Nanoparticle-Hydrogel Complexes for pH Monitoring

**DOI:** 10.3390/nano12224081

**Published:** 2022-11-20

**Authors:** Yeonjin Kim, Taeha Lee, Minsu Kim, Soojin Park, Jiashu Hu, Kyungwon Lee, Yoochan Hong, Insu Park, Gyudo Lee

**Affiliations:** 1 Department of Biotechnology and Bioinformatics, Korea University, Sejong 30019, Republic of Korea; 2 Interdisciplinary Graduate Program for Artificial Intelligence Smart Convergence Technology, Korea University, Sejong 30019, Republic of Korea; 3 Department of Medical Device, Korea Institute of Machinery and Materials (KIMM), Daegu 42994, Republic of Korea; 4 Department of Biomedical Engineering, Konyang University, Daejeon 35365, Republic of Korea

**Keywords:** polyaniline, hydrogel, ferrocene, pH monitoring, colorimetric nanoparticle, fast response, reversibility

## Abstract

Hydrogels containing redox-sensitive colorimetric nanoparticles (NPs) have been used to sense ambient pH in many fields owing to their simple and fast visualization capabilities. However, real-time pH monitoring still has limitations due to its poor response rate and irreversibility. Herein, we developed a fast responsive colorimetric hydrogel called ferrocene adsorption colorimetric hydrogel (FACH). Ferrocene, an organometallic compound, plays a vital role as an electron transfer mediator (i.e., redox catalyst) within the hydrogel network. FACH shows fast color change performance with high reactivity and penetrability to ambient pH changes. In detail, FACH shows distinct color change within 2 min under various pH conditions from four to eight, with good reliability. The speed for color change of FACH is approximately six times faster than that of previously developed colorimetric hydrogels, suggesting the fastest hydrogel-based colorimetric pH sensor. Furthermore, FACH shows reversibility and repeatability of the redox process, indicating scalable utility as a sustainable pH monitoring platform.

## 1. Introduction

pH is a representative indicator of the concentration of hydrogen ions in a solution and is used in a wide range of applications, including biosensors [1,2,3,4], food waste [5,6], pharmaceutical [7,8,9], biomedical [10,11], and environment [12,13,14]. Furthermore, various types of pH sensors have been developed, such as electrodes [15], transistors [16], cellulose paper [17,18], and optical devices [19,20]. Recently, hydrogels have attracted considerable attention in various application fields, including pH detection, due to their sensitive swelling property [21,22], flexibility [23], biodegradability [24,25,26], and biocompatibility [27,28,29,30]. In particular, research on colorimetric pH sensing using a hydrogel complex with colorimetric nanoparticles is being actively conducted. For example, Tamayol et al. combined alginate with nanobeads to measure the pH of the epidermis using flexible pH-sensing hydrogel fibers [31]. Liu et al. measured the pH of buffer solutions using chitosan hydrogel film composed of gold nanoparticle dimers [32]. Furthermore, Thakur et al. measured the pH of bacterial metabolism by fabricating a hydrogel film combining agarose hydrogel with polyaniline nanoparticles (PAni-NPs) [33]. Recently, Lee et al. advanced the PAni-NP-embedded hydrogels with high-throughput and large-area to measure the ambient pH of the cancer cells [34]. The aforementioned studies highlight the merits of the structural bonding of colorimetric NPs with polymeric hydrogel networks. This means that the manufacturing method is simple, the color change is intuitive, and it is easily adaptable to a variety of environments.

Despite these advantages, the pH sensing performance of hydrogel-based colorimetric sensors suffers from the low control stability and low responsiveness of the hydrogel [35]. For example, when a hydrogel is fabricated with a low concentration of polymer, the ionic transport rate of the hydrogel increases while its physical and mechanical stability reduces [36]. On the other hand, in the case of a high-density hydrogel, it is not possible to induce a uniform and fast response to changes in the ambient pH because the chemical reaction inside and outside the hydrogel is different [37]. In order to address this issue, it is suggested to use a redox catalyst that can improve the colorimetric reaction by mediating electron transfer [1]. Ferrocene, for example, can act as an effective redox catalyst through its intrinsic fast electron transfer rate and reversible redox reaction [38,39,40,41]. Based on these characteristics, ferrocene has been widely applied in various fields, such as optical devices [42], batteries [43], sensing [44], catalysts [45], and medicine [46]. In particular, it has already been used as a catalyst to improve colorimetric performance in redox-sensitive PAni-NPs-based sensors [1]. The role of ferrocene is to assist the proton-induced doping/de-doping process of PAni-NPs inside the hydrogel. PAni-NPs inside the hydrogel respond to the presence of H^+^ ions and exhibit colorimetric properties based on the doping/de-doping process. Since the H^+^ ion is a single cation, its gain and loss within the molecular structure of conductive polymer are directly related to the electron transfer (i.e., redox reaction). It has already been proven that ferrocene promotes electron transfer [38,39,40,41]. When the pH around the hydrogel decreases, the external H^+^ ion concentration increases, causing doping of PAni-NPs by internal diffusion of H^+^ ion. Conversely, when the pH increases, de-doping occurs in which H^+^ inside the PAni-NPs molecular structure escapes. In these processes, ferrocene can help electron transfer and promote doping/de-doping of PAni-NPs according to ambient pH.

In this study, we demonstrate the fabrication of a PAni-NP-hydrogel complex-based colorimetric pH monitoring platform with improved mechanical strength and rapid and reversible colorimetric response to ambient pH. The stability of the hydrogel was advanced by optimizing the fabrication conditions of the hydrogel. In addition, ferrocene was diffused into the hydrogel complex to prepare a ferrocene adsorption colorimetric hydrogel (FACH). The results show that the FACH exhibits rapid color change performance, high reactivity, and reversibility in response to the surrounding pH. We believe this advanced colorimetric hydrogel sensor (i.e., FACH) will be widely used in various industries, particularly biomedical and/or environmental, as an excellent pH monitoring platform.

## 2. Materials and Methods

### 2.1. Materials

Agarose, ammonium persulfate (APS), aniline (99%), distilled water (DW), ferrocene (98%), hydrochloric acid (HCl, 37%), pectin (extracted from apples, product number: 93854), phosphate-buffered saline (PBS), pure ethyl alcohol (99.5%), and Whatman 41 filter paper (grade 41) were purchased from Sigma-Aldrich (Burlington, MA, USA).

### 2.2. Synthesis of the PAni-NPs

The PAni-NPs were synthesized according to a previously reported method [34]. A base solution of 1.8 g pectin, 9 mL HCl (37%), 0.9 g aniline, and 50 mL of DW was prepared and then stirred at 23 °C. In order to produce uniform PAni-NPs, 25 mL of APS solution (91.2 mg/mL) was added dropwise to the base solution and then stirred for 4 h. After mixing 50% ethyl alcohol solution and PAni-NP solution, it was filtered with a desiccator and Whatman 41 filter paper to obtain PAni-NP of uniform size. The prepared PAni-NPs were diluted to 20 mg/mL for experiments.

### 2.3. Fabrication of the PAni-NP-Hydrogel Complex (PNHC)

In order to fabricate emeraldine salt (ES) state hydrogel complex, agarose was diluted in DW (pH 3) at a ratio of 5% and stirred at 23 °C. Then, it was put in a microwave oven and heated to dissolve the agarose completely. During this process, an appropriate temperature was maintained so that the agarose solution did not boil over. After cooling the completely dissolved agarose solution at 23 °C for 2 min, PAni-NP solution (pH 3, 20 mg/mL) was added in a 5:1 ratio and mixed by shaking. PAni-NPs-contained agarose solution (1 mL) was dispensed into a 6-well plate or petri dish (35 mm in diameter) to make 1 mm-thick PNHC. Finally, the solution was cured by cooling at 23 °C for 10 min on a flat surface to fabricate PNHC.

### 2.4. Fabrication of the FACH

In order to make a fast, responsive colorimetric hydrogel complex (i.e., FACH), 1 mL of ferrocene (4.5 mg/mL, pure ethyl alcohol) was dispensed on the completely cured PNHC surface and incubated for 30 s. After removing the ferrocene solution, 1 mL of PBS (pH 3) was dispensed, and the ferrocene residue on the PNHC surface was vigorously washed 5 times with a pipette. The same washing method was repeated 2 times. Then, the ferrocene residue on the wall of the well was carefully removed with an experimental cotton swab. The experiment was performed in a fume hood using 6-well plates.

### 2.5. Characterization

Images of PAni-NP solution, PNHC, and FACH were captured using a smartphone (Galaxy S21, Samsung, Korea), and their absorbance was analyzed using a hybrid multimode reader (Synergy H1, Agilent, Santa Clara, CA, USA). The pH of PAni-NPs solution, DW, and PBS was adjusted using a pH meter (Orion Star A211, Thermo Fisher Scientific, Waltham, MA, USA).

## 3. Results and Discussion

### 3.1. Fabrication of Hydrogel Complex

**Figure 1:**
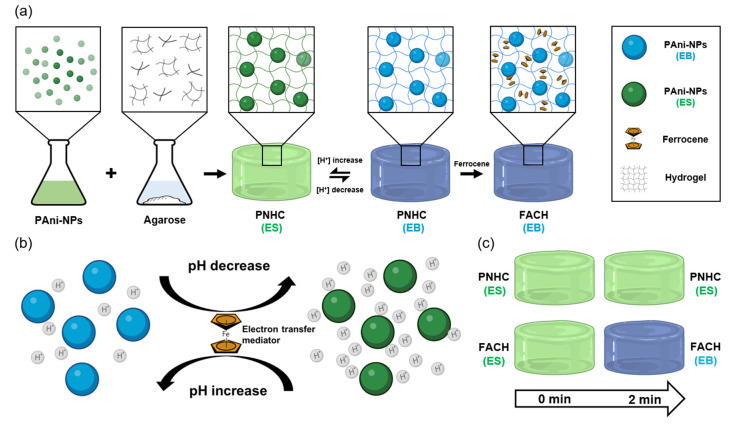
(**a**) Schematic illustration of PNHC and FACH fabrication processes and working principle. (**b**) The role of ferrocene as an electron transfer mediator, accelerating the change color of hydrogel complex. (**c**) Comparison of color change between PNHC and FACH after high pH treatment within 2 min.

The hydrogel complex was fabricated by following the protocol established in a previous study [34]. Briefly, PNHC was prepared by mixing the PAni-NP solution and agarose solution. The size of PAni-NPs was 84.0 ± 2.2 nm (mean ± standard deviation), which was measured by atomic force microscopy. Subsequently, PNHC was incubated in ferrocene solution for 30 s to produce a fast, responsive hydrogel complex (i.e., FACH) (Figure 1a). Within FACH, ferrocene act as a mediator of redox reactions to transfer electrons within the hydrogel meshes to PAni-NPs (Figure 1b). This effect of ferrocene to increase the electron transfer efficiency plays a vital role in the process of color change in FACH. Our hydrogel complex based on PAni-NPs visually informs the changes in ambient pH based on two representative colors, green (ES) and blue (emeraldine base, EB), in a specific pH range. In order to compare the color changes of PNHC and FACH over time, both types of hydrogels were treated with high pH solution for 2 min (Figure 1c). At the given time, FACH showed a distinct color change to blue (EB), whereas PNHC remained green (ES).

### 3.2. Optical Properties of PAni-NP Solution

**Figure 2:**
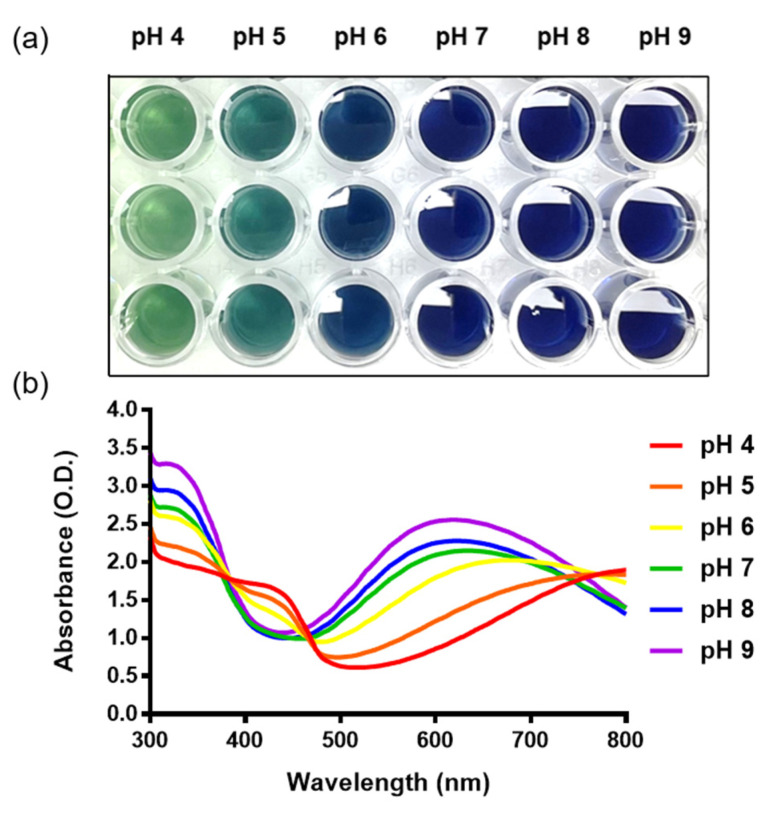
(**a**) Real image of Pani-NP solution by pH in a 96-well plate. (**b**) Absorbance spectra of Pani-NP solutions at different pH values.

In order to investigate the colorimetric properties of PAni-NP solution as a function of pH level, optical images were taken for visual color comparison. It was confirmed that the color of the Pani-NP solution gradually changed from green (ES state) to blue (EB state) as pH increased (Figure 2a). The color of the Pani-NP solution for each pH is vivid enough to be visually distinguished, which is consistent with the color change of Pani-NPs reported previously [34].

Figure 2b shows the absorbance of the Pani-NP solution for each pH level. The absorbance peak corresponding to the π–π* transition of the benzenoid ring and the polaron band was analyzed using a spectrophotometer. Pani-NP solutions were prepared at various pH levels (from 4 to 9). As the pH increased, the absorbance at 430 nm tended to decrease, and the absorbance at 600 nm increased. These features of the absorbance spectra indicate the high quality of Pani-NPs, which is consistent with the previous study [34].

### 3.3. Optimization of the PNHC and Fabrication of the FACH

**Figure 3:**
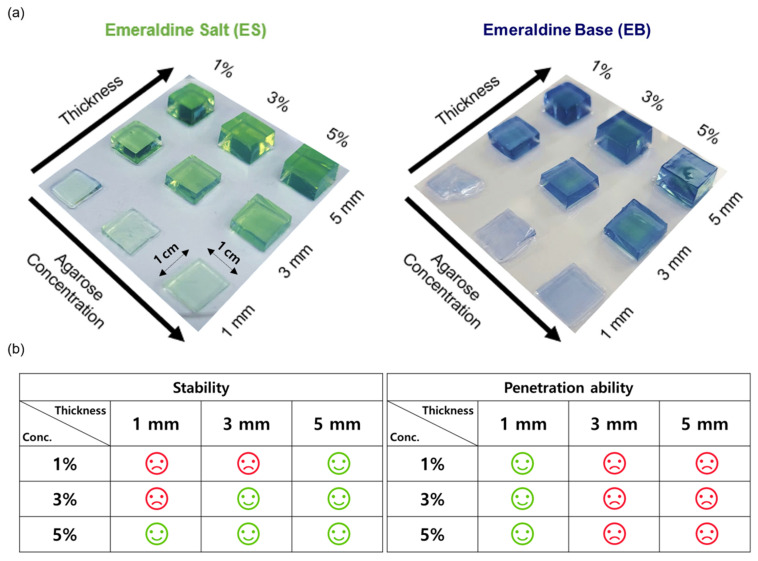
(**a**) Real images of ES state and EB state PNHC according to thickness and agarose concentration. The hydrogel images in the right panel were taken after 15 min treatment with a PBS solution (pH 10). (**b**) PNHC optimization table considering stability and penetration ability (1 mm of thickness and 5% of agarose concentration were optimized).

To seek the optimal condition for satisfying both the mechanical stability and colorimetric response ability of PNHC, nine different types of PNHC were created by combining various agarose concentrations (1, 3, and 5%) and thicknesses (1, 3, and 5 mm). The green PNHC (ES state) was uniformly cut to a size of 1 × 1 cm^2^, and the color change was observed by aliquoting PBS (pH 10) (Figure 3a). As a result, the 1 mm-thick PNHC lost its shape due to low gel stability except for 5% agarose concentration, making it unsuitable for additional experiments. In the case of 3 mm and 5 mm thick PNHC, it was confirmed that the color inside the hydrogel did not wholly change to blue at all gel concentrations for a given time (15 min) and remained green (ES state). These nonuniform colorimetric properties are attributable to low penetration ability due to the hydrogel thickness, which is unsuitable for a rapidly responsive colorimetric hydrogel platform. Therefore, among the nine different types of Pani-NP-hydrogel complexes, PNHC with an agarose concentration of 5% and a thickness of 1 mm was finally selected in terms of the highest stability and colorimetric response-ability (Figure 3b). In the subsequent experiments, the hydrogel volume was carefully calculated to keep the identical thickness of 1 mm (see Section 2.3), and then, FACH was newly fabricated by adsorbing ferrocene to the optimized PNHC (see Section 2.4).

### 3.4. Comparison of Colorimetric Response and Sensitivity between PHNC and FACH

To observe the effect of ferrocene treatment on PNHC from various perspectives, we investigated the relationship between colorimetric response and penetration rate of ferrocene according to the ferrocene treatment time (5, 30, 60, 120, and 300 s). Figure S2 shows no difference in the reaction rate of FACH according to the ferrocene treatment time from 5 to 120 s. This means that the penetration of ferrocene took place within 5 s. When the treatment time was over 120 s, the reaction rate was somewhat decreased, possibly due to the hydrogels’ blocking pores by ferrocene residues. The color of the FACH was hardly changed with ferrocene treatment for up to 120 s. However, it turned slightly yellow when processed for 300 s. This is because ferrocene residues were left on the hydrogel surface and network due to long-term treatment, and they were not removed even by strong washing. Therefore, in order to maintain the transparency of the FACH, it is recommended to treat the ferrocene within 120 s. As such, by controlling the concentration and treatment time of ferrocene, PNHCs with various shapes and sizes can be fabricated into FACHs.

**Figure 4:**
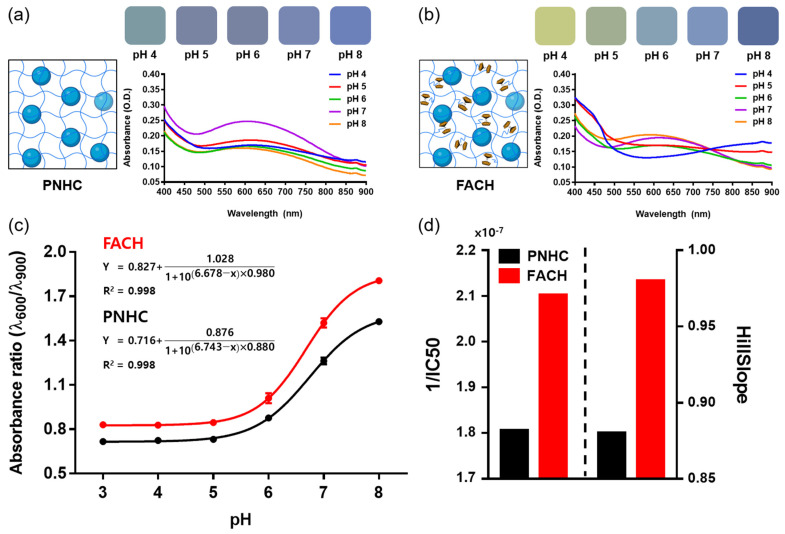
PNHC and FACH were fabricated with 1 mm-thick 5% agarose gels and treated with PBS solution (pH 4, 5, 6, 7 and 8) for a sufficient time (1 h) for chemical reactions to occur, respectively. FACH was fabricated by adsorbing ferrocene to PNHC. Colors of (**a**) PNHC and (**b**) FACH according to pH and the corresponding absorbance spectra are presented. (**c**) Absorbance ratio (λ_600_/λ_900_) of PNHC and FACH with pH levels. The data were obtained by quadruplicate measruements (Mean ± SD). (**d**) Comparison of HillSlope and 1/IC50 between PNHC and FACH.

In order to compare the performance of PNHC and FACH as pH monitoring platforms, colorimetric responses were identified at each pH level. In particular, the actual color of PNHC was obtained in the pH 4 to 8 range. PNHC showed cyan at low pH and blue at high pH, but the color change was relatively insignificant compared to the PAni-NP solution (as shown in Figure 2a). The color change of these PNHCs was quantified by spectrophotometric analysis. As a result, the increase or decrease in the absorbance spectrum according to pH was not apparent, and it was difficult to distinguish the spectrum in a given pH range (Figure 4a). This is due to the slight color change of PNHC with the pH change caused by slow electron transfer in the absence of ferrocene molecules.

In contrast with PNHC, FACH exhibits a more apparent color change for each pH, and so do the corresponding absorbance spectra (Figure 4b). The quantification method for evaluating the FACH’s performance was the same as that of PNHC, and as a result, FACH showed a bright green color at low pH and a vivid blue color at high pH. In summary, the colorimetric responses of FACH were much more distinctive than those of PNHC. A similar trend was observed in the absorbance spectrum of FACH: as the pH increased, the absorbance intensity increased at the 600 nm wavelength but decreased at the 900 nm wavelength. The spectral properties of FACH according to pH are relatively worse than that of the PAni-NP solution, which is attributed to the enormous light scattering on the hydrogel in spectrophotometry.

Next, we measured the color change efficiency of PHNC and FACH according to the pH and compared the absorbance ratio (λ_600_/λ_900_) (Figure 4c and Supplementary Information Figure S1). The absorbance ratio for both PNHC and FACH showed a sigmoidal conformation for pH levels from 3 to 8 and was fitted to the sigmoidal model below.

(1) $$\mathrm{Absorbance}\;\mathrm{ratio}\;(\lambda_{600}/\lambda_{900})=\mathrm{Bottom}+\frac{\left(\mathrm{Top}-\mathrm{Bottom}\right)}{{1+10}^{\left(\mathrm{LogIC}50-\mathrm{pH}\right)\times \mathrm{HillSlope}}}$$

where the Top and Bottom are the plateaus in the same units as Y. HillSlope denotes the steepness of the curve family. IC50 is the half maximal inhibitory concentration, indicating the pH value required to bring the curve down to a point halfway between the Top and Bottom plateaus of the curve. For R^2^, both PNHC and FACH were excellent (R^2^ = 0.998). We then compared 1/IC50 and HillSlope of PNHC and FACH (Figure 4d). These two values are related to the sensitivity of the colorimetric hydrogels: the sensitivity is proportional to the values of HillSlope and 1/IC50. The HillSlope of PNHC and FACH were 0.880 and 0.980, respectively, which improved by only 11% in FACH. The 1/IC50 for PNHC and FACH was 1.8 × 10^−7^ and 2.1 × 10^−7^, respectively, indicating that FACH was approximately 17% more sensitive than PNHC. The colorimetric efficiency of FACH has slightly improved compared to PNHC. However, the colorimetric responses for PNHC and FACH were similar for a long-term chemical reaction (1 h). It is reasonable because the same type of NPs (i.e., polyaniline) was embedded in both PNHC and FACH. It also implies that the addition of ferrocene does not affect the colorimetric properties of the NPs inside the hydrogel complex. It is questionable how the color change of FACH is more distinct than that of PNHC.

### 3.5. Colorimetric Kinetics of FACH in pH Sensing

**Figure 5:**
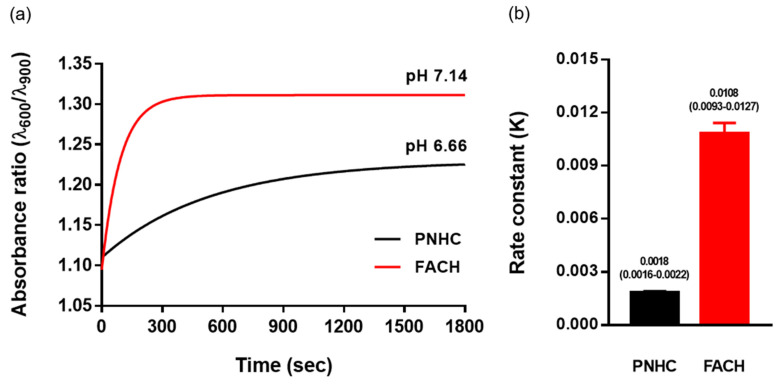
(**a**) Change of absorbance ratio (λ_600_/λ_900_) over time after treatment with PBS (pH 8) in PNHC (black) and FACH (red). (**b**) Comparison of rate constants (K = τ^−1^) between PNHC and FACH.

To investigate the colorimetric kinetics of FACH and PNCH, we measured the colorimetric response times. Colorimetric kinetics is defined as a color shift over a relatively short period under rapid changes in the ambient pH level. To this end, pH 3-conditioned PNHC and FACH were treated with a high pH solution (2 mL, pH 8, PBS), and absorbance over time was measured using a spectrophotometer. As a result, in the case of FACH, the reaction was almost completed within 2 min (tau = 91.98), and at the end of the reaction, the absorbance ratio (λ_600_/λ_900_) of PNHC and FACH reached 1.23 and 1.32, respectively (Figure 5a). The values 1.23 and 1.32 can be converted into pH 6.66 and 7.14 by following the PNHC model established in Figure 4c. The discrepancy between the saturated pH level and the treated pH level is due to the dilution of the H^+^ concentration as the solution inside the hydrogel complex mixed with the externally supplied solution. We calculated the rate constant (K = τ^−1^) values of PNHC and FACH by fitting the exponential equation to both kinetic curves. In the case of the kinetic curve of PNHC does not appear to have reached saturation, but it fits the exponential model equation well, and its rate constants were calculated from curve fitting and displayed in Figure 5b. This indicates that there are sufficient data points for the kinetics of PNHC and that more data points at longer times are not essential. The K value (mean, 95% confidence interval) of FACH (0.0108, 0.0093–0.0127) was found to be approximately six times higher than that of PNHC (0.0018, 0.0016–0.0022) (Figure 5b). As a result, it was confirmed that FACH responds faster and more sensitively to changes in pH than the conventional PNHC.

### 3.6. Reversible Test Using FACH and Its Applications

**Figure 6:**
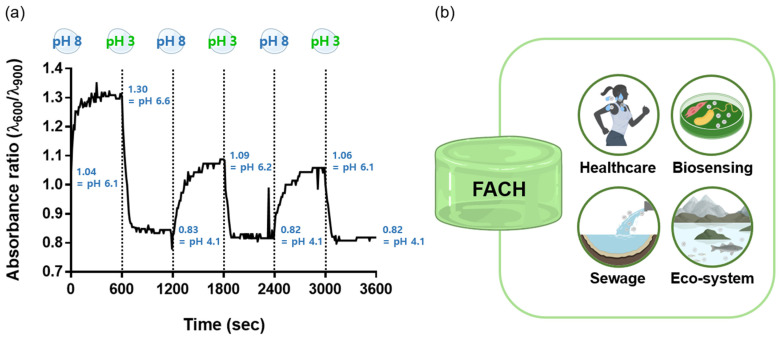
(**a**) Reversible test of FACH through recording absorbance ratio (λ_600_/λ_900_) under consecutive pH changing conditions with 10 min-intervals. (**b**) Applicable fields of FACH.

A reversible test was conducted to investigate the possibility of using FACH as a pH monitoring platform. Changes in the absorbance ratio (λ_600_/λ_900_) of FACH were measured at 10 min intervals by treating low-pH PBS and high-pH PBS (2 mL in volume). The starting FACH was conditioned in a pH 3 state. As shown in Figure 6a, a reversible colorimetric response of FACH was confirmed. The absorbance ratio of FACH responds to the treated solution and changes repeatedly and rapidly over time. However, the magnitude of the absorbance ratio tends to decrease after the first cycle, which is due to the property that the FACH retains water therein and is an explainable phenomenon. In detail, the reversible test started with a FACH (1 mL) having pH 3. Subsequently, we treated 2 mL of a pH 8 solution on the FACH. During the reaction, the solution inside the FACH meets the external solution, resulting in a change in the final pH of the solution inside the FACH. This could be described by the following calculation process based on algebra, and the result is similar to the pH value we measured, as follow:

(2) $$\mathrm{Hydrogel}\;\left(\mathrm{pH}\right)=\frac{\mathrm{pH}\;3}{1\;\mathrm{mL}}+\left(\frac{\mathrm{pH}\;8}{1\;\mathrm{mL}}+\frac{\mathrm{pH}\;8}{1\;\mathrm{mL}}\right)=\frac{\mathrm{pH}\;6.33}{1\;\mathrm{mL}}\approx \;\mathrm{pH}\;6.6\;\left(measured\right)$$

Afterward, we treated 2 mL of a pH 3 solution on the FACH (pH 6.6 and 1 mL). By the same mechanism, the pH of the hydrogel is determined.

(3) $$\mathrm{Hydrogel}\;\left(\mathrm{pH}\right)=\frac{\mathrm{pH}\;6.6}{1\;\mathrm{mL}}+\left(\frac{\mathrm{pH}\;3}{1\;\mathrm{mL}}+\frac{\mathrm{pH}\;3}{1\;\mathrm{mL}}\right)=\frac{\mathrm{pH}\;4.2}{1\;\mathrm{mL}}\approx \;\mathrm{pH}\;4.1\;\left(measured\right)$$

The slight discrepancy between predicted pH and measured pH is from the difference between the doping and de-doping rate of the PAni-NPs in hydrogels. In detail, this is due to the difference in required energies for the doping/de-doping state of PAni-NPs in the hydrogels. At low pH conditions, the H^+^ ion binds to the PAni-NPs (i.e., doping state). However, at high pH conditions, the H^+^ ions desorb from PAni-NPs (i.e., de-doping state). At this time, the de-doping process occurs slowly compared to the doping process. This is because the energy required to break the existing ionic bond between H+ ions and PAni-NPs is greater than the energy required for H+ ions to bind to PAni-NPs. This interpretation can explain why the hydrogel takes longer to reach an equilibrium state when the ambient pH increases than when it decreases. In this way, the redox reaction of FACH is repeated in the reversible test. However, a small loss of ferrocene inside FACH is accumulated during the repeat experiments. This implicates improvement of FACH is needed, including covalent bonding of ferrocene on the hydrogel networks.

FACH is a stable hydrogel-based biosensor that can visually confirm the color change according to the pH level just by adhering to human skin or using it as a bacterial culture solid medium [34]. In addition, the colorimetric reaction can be confirmed even in an aquatic environment such as a sewage treatment plant or a river. Based on these results, we propose that FACH can be applied to various fields requiring pH monitoring, such as healthcare, biosensing, sewage, and the ecosystem (Figure 6b). It would be useful to photograph or record the FACH over time to analyze the color change. Furthermore, more accurate pH sensing performance can be expected by applying machine learning to analyze the color of FACH.

## 4. Conclusions

In this study, we optimized the physical stability, penetrability, and reactivity of colorimetric NP-hydrogel complexes for pH detection and monitoring. In detail, we developed a fast, responsive, reversible colorimetric hydrogel complex (called FACH) by adsorbing ferrocene on the PNHC. FACH showed an excellent color change at various pH levels (from 4 to 8). For long-term (1 h) reactions, the colorimetric responses for FACH were similar to those of the PNHC. This indicates the effect of redox-sensitive PAni-NPs as well as the stability of the redox reaction in the hydrogel complex in the absence and presence of ferrocene. Importantly, FACH showed an approximately 6-fold increase in response rate than PNHC in colorimetric kinetics. In addition, it was found that FACH has reversibility and repeatability. Finally, this advanced pH monitoring platform based on FACH can be used in many applications, including in biomedical and environmental industries.

## Supplementary Materials

The following supporting information can be downloaded at: https://www.mdpi.com/article/10.3390/nano12224081/s1, Figure S1. Absorbance ratio (λ_600_/λ_900_) including pH 0 and 14 conditions of (a) PNHC and (b) FACH. Hy-drogels were treated with various pH solutions for a sufficient time (1 h) for chemical reactions; Figure S2. Measurement of FACH reaction rate according to ferrocene treatment time (5, 30, 60, 120, and 300 s). To the pH 3 hydrogels treated with ferrocene for different times, 2 mL of PBS (pH 8) was added, and the absorbance ratio (λ_600_/λ_900_) was observed for 30 min.
