# Supplementary material for: Fast Responsive, Reversible Colorimetric Nanoparticle-Hydrogel Complexes for pH Monitoring
Source: Nanomaterials (Basel). 2022 Nov 20;12(22):4081. doi: 10.3390/nano12224081 (PMC9699376; doi:10.3390/nano12224081)
Supplement: Supplementary file 1 [file nanomaterials-12-04081-s001.zip › nanomaterials-1968225-supplementary.pdf]

nanomaterials

Supplementary Information

Fast Responsive, Reversible Colorimetric Nanoparticle-Hydrogel Complexes for pH Monitoring

Yeonjin Kim [1,†], Taeha Lee [1,2,†], Minsu Kim [1], Soojin Park [1], Jiashu Hu [1], Kyungwon Lee [1], Yoochan Hong [3,*], Insu Park [4,*] and Gyudo Lee [1,2,*]

1 Department of Biotechnology and Bioinformatics, Korea University, Sejong 30019, Republic of Korea
2 Interdisciplinary Graduate Program for Artificial Intelligence Smart Convergence Technology, Korea University, Sejong 30019, Republic of Korea
3 Department of Medical Device, Korea Institute of Machinery and Materials (KIMM), Daegu 42994, Republic of Korea
4 Department of Biomedical Engineering, Konyang University, Daejeon 35365, Republic of Korea
* Correspondence: ychong1983@kimm.re.kr (Y.H.); insupark@konyang.ac.kr (I.P.); lkd0807@korea.ac.kr (G.L.)
† These authors contributed equally to this work.

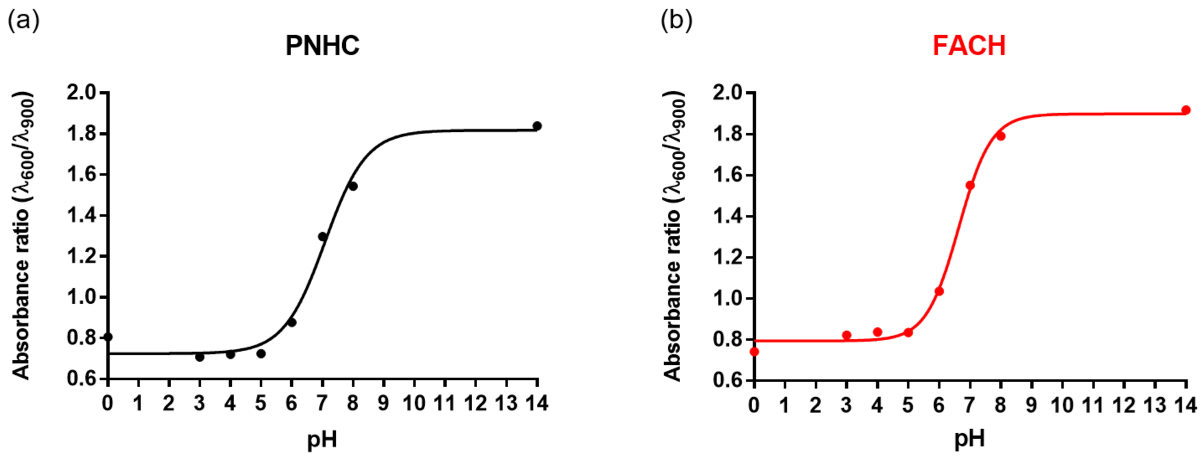

Figure S1. Absorbance ratio ($\lambda_{600}/\lambda_{900}$) including pH 0 and 14 conditions of (a) PNHC and (b) FACH. Hydrogels were treated with various pH solutions for a sufficient time (1 h) for chemical reactions.

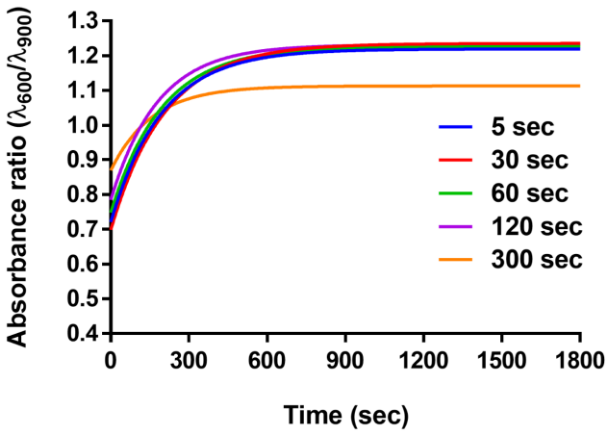

Figure S2. Measurement of FACH reaction rate according to ferrocene treatment time (5, 30, 60, 120, and 300 s). To the pH 3 hydrogels treated with ferrocene for different times, 2 mL of PBS (pH 8) was added, and the absorbance ratio ($\lambda_{600}/\lambda_{900}$) was observed for 30 min.
